# Memantine has a nicotinic neuroprotective pathway in acute hippocampal slices after an NMDA insult

**DOI:** 10.1016/j.tiv.2022.105453

**Published:** 2022-08-06

**Authors:** Yancy Ferrer-Acosta, Sergio Rodriguez-Massó, Dinely Pérez, Vesna A. Eterovic, P.A. Ferchmin, Antonio Henrique Martins

**Affiliations:** a Department of Neuroscience, Universidad Central del Caribe, Laurel Avenue 2U6, Lomas Verdes, Bayamón 00956, Puerto Rico; b Department of Pharmacology and Toxicology, University of Puerto Rico, Medical Sciences Campus, Los Paseos Avenue, Guillermo Arbona Building, San Juan 00935, Puerto Rico; c Department of Biochemistry, Universidad Central del Caribe Laurel Avenue, #100, Santa Juanita, Bayamón 00956, Puerto Rico; d Neuroprotection for Life, 480 E Village Dr., Carmel, IN 46032, USA

**Keywords:** Memantine, Neuroprotection, 4R-cembranoid, Cholinergic mechanism

## Abstract

Memantine is a non-competitive antagonist with a moderate affinity to the *N*-methyl-D-Aspartate (NMDA) receptor. The present study assessed memantine’s neuroprotective activity using electrophysiology of ex-vivo hippocampal slices. Interestingly, a nicotinic component was necessary for memantine’s neuroprotection (NP). Memantine demonstrated a bell-shaped dose-response curve of NP against NMDA. Memantine was neuroprotective at concentrations below 3 μM, but the NP declined at higher concentrations (*>*3 μM) when memantine inhibits the NMDA receptor. Additional evidence that memantine NP is mediated by an alternate mechanism independent of the inhibition of the NMDA receptor is supported by its ability to protect neurons when applied before or after the NMDA insult and in the presence of D(−)-2-Amino-5-phosphonopentanoic acid (APV), the standard NMDA receptor inhibitor. We found several similarities between the memantine NP mechanism and the neuroprotective nicotinic drug, the 4R cembranoid. Memantine’s NP requires the release of acetylcholine, the activation of α4β2, and is independent of MEK/MAPK signaling. Both 4R and memantine require the activation of PI3K/AKT for NP against NMDA-mediated excitotoxicity, although at different concentrations. In conclusion, our studies show memantine is neuroprotective through a nicotinic pathway, similar to the nicotinic drug 4R. This information leads to a better understanding of memantine’s mechanisms of action and explains its dose-dependent effectiveness in Alzheimer’s and other neurological disorders.

## 1. Introduction

Alzheimer’s disease (AD) is a neurological disorder pathologically characterized by two protein aggregates, β-amyloid plaques and tau neurofibrillary tangles, associated with severe cognitive impairment. One of the circuits most affected by this disease is the basal cholinergic system of the forebrain, which includes the entorhinal, limbic, and cortical areas of the brain. Treatment for early to moderate AD symptoms, which include mild cognitive impairment, is reversible anticholinergic drugs such as donepezil, galantamine, or rivastigmine. For moderate to late stages of AD with severe cognitive impairment, memantine, an antagonist of *N*-methyl-D-aspartate (NMDA)- receptors, is used alone or combined with acetylcholinesterase inhibitors (Rogawski and Wenk, 2003; Robinson and Keating, 2006).

Memantine is an amantadine derivative that elicits neuroprotection (NP) in humans. It is a non-competitive (open channel) antagonist with a low to moderate affinity for NMDA receptors (Rammes et al., 2008), and it is believed that its principal mechanism of action is antagonism of NMDA receptors (Johnson and Kotermanski, 2006). However, memantine is not selective and interacts with other receptors such as 5-HT3 (Reiser et al., 1988)) and nicotinic receptors (Parsons et al., 1999). Studies have shown that memantine has a higher affinity for alpha 7 nicotinic acetylcholine receptors (α7 nAChR) than for NMDA receptors (Aracava et al., 2005). At concentrations >10 μM, memantine is a potent α4β2 nicotinic inhibitor; interestingly, other NMDA antagonists such as MK-801 also block this receptor, albeit with less potency than memantine (Ferchmin et al., 2005; Buisson and Bertrand, 1998).

The cembranoid [(1S, 2E, 4R, 6R, 7E, 11E)-cembra-2,7,11-triene-4,6-diol] (4R) is a comparative nicotinic-receptor ligand with additional insecticidal, antifungal, antimicrobial, and antiviral properties (Aqil et al., 2011; Yan et al., 2019). For this study, 4R was purified from tobacco leaves (El Sayed et al., 2008; Wahlberg and Eklund, 1992; Eklund et al., 1992). Our group (Eterović et al., 2011, 2013) has found that tobacco cembranoids, specifically the stereoisomer 4R, show neuroprotective characteristics. The 4R cembranoid can significantly decrease NMDA-induced excitotoxicity in animals and ex vivo models of organophosphate poisoning, ischemic stroke, and Parkinson’s disease (Ferchmin et al., 2003, 2013, 2014; Martins et al., 2015; Ferchmin et al., 2005; Hu et al., 2017). In patch-clamp studies, 25 μM of 4R reversibly inhibited acetylcholine (ACh) evoked currents in an α7 nAChR-transfected neuroblastoma cell line. Interestingly, the classical α7 nAChR antagonist methyllycaconitine (MLA) also blocks NMDA-induced excitotoxicity using the same pharmacological pathway as 4R in rat hippocampal slices (Ferchmin et al., 2013). These results indicate that the observed neuroprotective effect can be attributed to inhibition of the α7 nAChR. Previous studies have shown that the 4R neuroprotective signaling route uses the phosphatidylinositol 3-kinase/protein kinase B (PI3K/AKT) cell survival-promoting pathway and not the Mitogen-activated protein kinase / extracellular signal-regulated kinases (MAPK/ERK) pathway (Ferchmin et al., 2005).

Although the literature shows some findings of the nicotinic neuroprotective pathway of memantine (Maskell et al., 2003), our work completes and expands this new mechanism of action, leading to a better understanding of this molecule used to treat moderate to severe Alzheimer’s disease symptoms.

## 2. Materials and methods

### 2.1. General materials

Dimethyl sulfoxide (DMSO), memantine, *N*-methyl-D-aspartate (NMDA), D (−)-2-Amino-5-phosphonopentanoic acid (APV), vesamicol, nifedipine, dihydro-β-erythroidine hydrobromide (DHβE), [1α,4 (S),6β,14α,16β]-20-Ethyl-1,6,14,16-tetramethoxy-4-[[[2-(3-methyl-2,5-dioxo-1-pyrrolidinyl)benzoyl]oxy]methyl]aconitane-7,8-diol citrate salt (MLA), PD98059, LY294002 were obtained from Sigma-Aldrich (St. Louis, MO). Cembranoid (1S,2E, 4R, 6R,7E,11E) -cembra-2,7,11-triene-4,6-diol (4R) (CAS number 57605–81-9) was prepared and purified from tobacco by Prof. K. El Sayed (School of Pharmacy, University of Louisiana, Monroe, LA).

### 2.2. Electrophysiological recordings

Male Sprague Dawley rats (120–200 g) from the Universidad Central del Caribe (UCC) colony were used to prepare hippocampal slices in agreement with the NIH. The authors certify that the use of animals was carried out in accordance with the National Institute of Health Guide for the Care and Use of Laboratory Animals (NIH Publications No. 80–23) revised in 1996 and institutional policies of UCC, and it is available upon request.

The detailed ex vivo hippocampal slices electrophysiology method is described by (Eterović et al., 2011). Briefly, approximately 30 slices of 400 μm from the hippocampi of two rats were distributed equally among three lanes of an incubation chamber. For dissection and incubation of slices in the chamber, an artificial cerebrospinal fluid (ACSF) solution containing 125 mM NaCl, 3.3 mM KCl, 1.25 mM NaH_2_PO_4_, 2 mM MgSO_4_, 2 mM CaCl_2_, 25 mM NaHCO_3_, and 10 mM glucose was used. After dissection, the hippocampi were left to stabilize in ACSF for 1 h before measuring the initial population spike (PS). A maximum of seven slices per lane were analyzed for each individual experiment, and, on average, 21 slices were tested for each experimental condition because the ex vivo chamber consists of three experimental lanes. Approximately one hour after dissection, the Schaffer collaterals were stimulated with an electric stimulus twice the strength required to synaptically elicit a threshold PS from the pyramidal neurons of the CA1 area. Before any pharmacological treatment, the initial PS recorded from each slice (recorded as PS area = msec × mV) was considered the 100% response for that slice. The percent decrease in the PS area after NMDA treatment was the measure of neuronal injury. In contrast, the percentage recovery of PS after NMDA by pre- or post-application of a neuroprotective compound was considered a measure of neuroprotection (Eterović et al., 2011). The excitotoxic insult to the slices was caused by superfusion with 0.5 mM NMDA for 10 min in ACSF. NMDA was washed out for 1 h. Subsequently, each slice was electrically stimulated in the same area and with the same intensity that was used to determine the initial PS. The final PSs were compared with the initial PS, and the percentage of initial response remaining at the end of the experiment was a measure of slice recovery. PSs are the sum of axon potentials that are all-or-none responses; therefore, the decrease of population spikes measures the decline of the number of neurons capable of producing axon potentials, and thus it is an early expression of apoptosis (Andersen et al., 1971; Martins et al., 2012, 2015). The experimental conditions were set to allow an average of 20% recovery of PSs in controls (Ferchmin et al., 2000). In each experiment, there were three conditions: control (only with an insult) and two with drugs. Some drugs were dissolved in DMSO 0.1% (4R, vesamicol), others in nanopure water (memantine, nifedipine, DHβE, and MLA). DMSO did not affect PS recovery at the concentrations used (0.1% *v*/v), and all groups had the same exposure to DMSO when required.

### 2.3. Statistical analyses

Data were plotted in SigmaPlot 12.0 by SYSTAT California. One-way analysis of variance (ANOVA) was used when the data had a normal distribution. In several experiments in which the hippocampal slices did not recover after NMDA treatment, the data failed the normality test. The less robust, nonparametric Kruskal-Wallis one-way ANOVA on ranks test was used in these cases. Tukey was the post -hoc test, which is more conservative than the Student-Newman-Keuls test when (n) is the same for all experiments; otherwise, the Dunn’s test was used. Data are shown as box plots showing the minimum, first quartile, median, third quartile, and maximum data value. Dots beyond the whiskers in the box plot represent the outliers. A minimum of 7 slices from two different animals were used for each point.

## 3. Results

### 3.1. The neuroprotective effect of memantine is dose-dependent

**Fig. 1.**
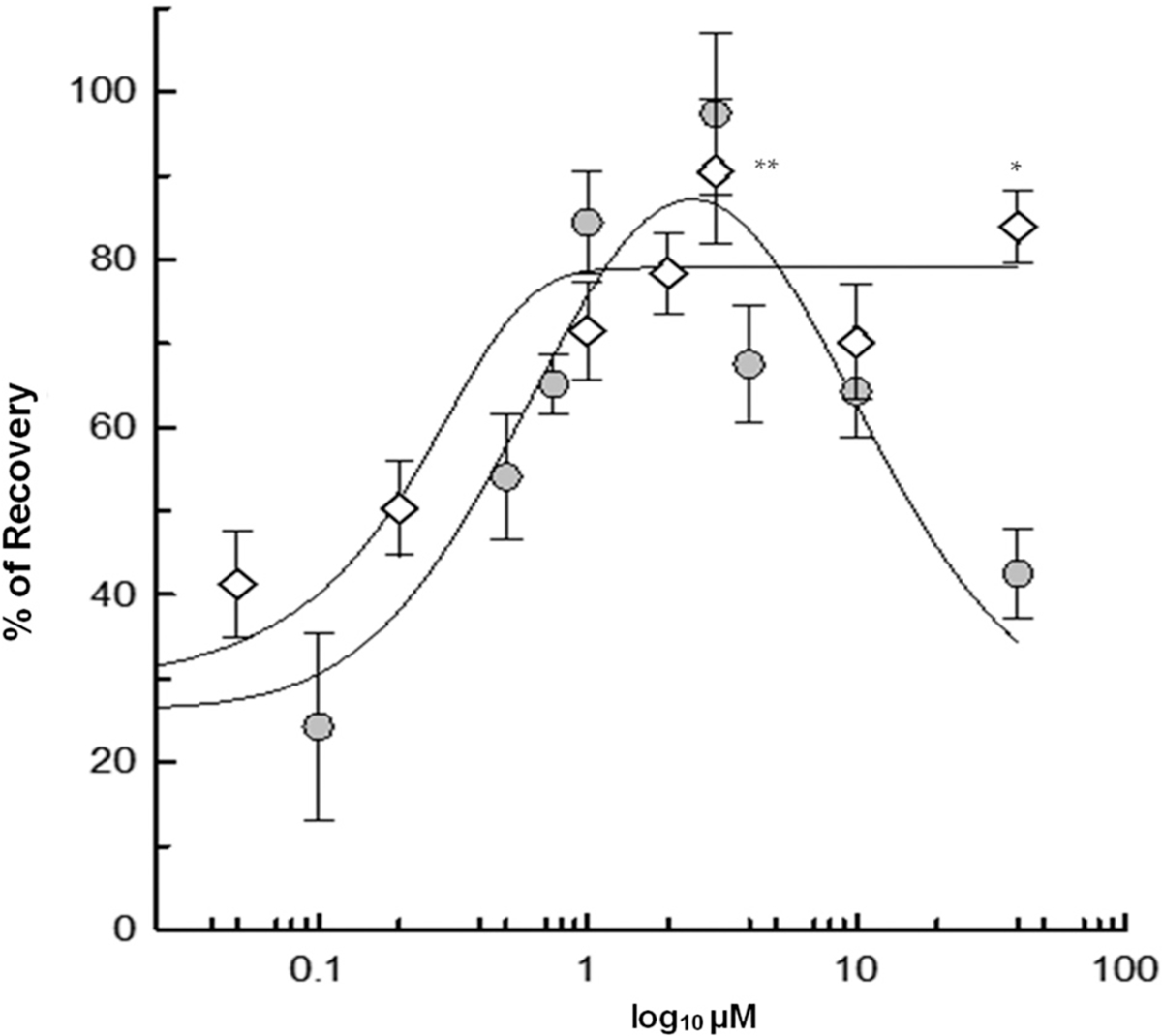
The neuroprotection curves for memantine and 4R. A dose-response curve against NMDA excitotoxicity was conducted to compare 4R and memantine potency and efficacy in neuronal recovery. Excitotoxicity was assessed by superfusion of 0.5 mM NMDA for 10 min using ex vivo hippocampal slices. 4R (diamonds) or memantine (gray circles) were superfused in a dose-dependent manner 1 h after the NMDA insult. In this figure, the Y-axis shows the % recovery of population spikes after NMDA insult, and X-axis shows the log10 concentrations of 4R and memantine in a dose-response curve. One-way ANOVA analysis showed that at 1 μM, 3 μM, and 10 μM, memantine and 4R are neuroprotective. 4R at 3 μM and 40 μM was significantly more neuroprotective than memantine at 40 μM (**p = 0.0059 and *p = 0.0158, respectively). Results were obtained with a minimum of 7 slices from two animals per point. See text for the number of hippocampal slices used in each point.

A dose-response curve was constructed to determine the concentrations of memantine and 4R required for optimal neuroprotection against NMDA-induced excitotoxicity using ex vivo rat hippocampal slices. Slices were superfused with 0.5 mM NMDA for 10 min as the excitotoxic insult. Fig. 1 shows the effect of 4R and memantine added 1 h after NMDA on the hippocampal slices’ population spike (PS) recovery. After the insult, control slices recovered 22.5 ± 3.45%, *n* = 154. Increasing 4R concentrations up to 40 μM induced a significant recovery in PS (neuroprotection): 0.2 μM 50.3 ± 5.6% *n* = 28; 1 μM 71.48 ± 5.92 *n* = 21; 2 μM 78.40 ± 4.86% *n* = 49, 3 μM 90.51 ± 8.65% n = 21; 10 μM 70.17 ± 6.89% n = 21; 40 μM 83.87 ± 4.34 *n* = 42, showing a wide neuroprotective range. Increasing amounts of memantine resulted in a distinctive bell-shaped curve, 0.75 μM 65.1 ± 3.6% *n* = 14, 1 μM 84.39 ± 6.15% n = 21; 3 μM 97.37 ± 9.57% *n* = 7, 4 μM 67.48 ± 7.03% n = 7; 10 μM 64.25 ± 5.49% n = 14; 40 μM 42.52 ± 5.41% *n* = 7. A dose comparison between 4R and memantine using a one-way ANOVA shows that at 1 μM, 3 μM, and 10 μM, memantine and 4R are both neuroprotective, and there is no significant difference between their % recovery (n.s. *p* = 0.841, *p* = 0.999, and p = 0.999, respectively). Nevertheless, at 40 μM, memantine’s neuroprotection significantly decreases compared to its lower doses of 1 μM and 3 μM (*p = 0.0278 and *p = 0.0136, respectively). At 40 μM, 4R’s neuroprotection showed no significant difference compared to its lower doses of 1 μM and 3 μM (n.s. *p* = 0.758, *p* = 0.989, respectively). In contrast, 4R at 40 μM showed a significantly higher % recovery than memantine at the same dose (*p = 0.0158).

The concentrations between 1 and 3 μM of memantine induced maximum neuroprotection in hippocampal slices. Therefore, the lowest concentration of memantine that achieved maximum neuroprotection, 1 μM, was chosen for further studies. Memantine at 1 μM is also the minimal effective concentration found in the plasma of treated patients and is close to the therapeutically relevant 0.9 μM found in brain extracellular fluid (Rammes et al., 2008; Robinson and Keating, 2006; Rogawski and Wenk, 2003).

### 3.2. Memantine can prevent and revert NMDA-induced excitotoxicity

**Fig. 2.**
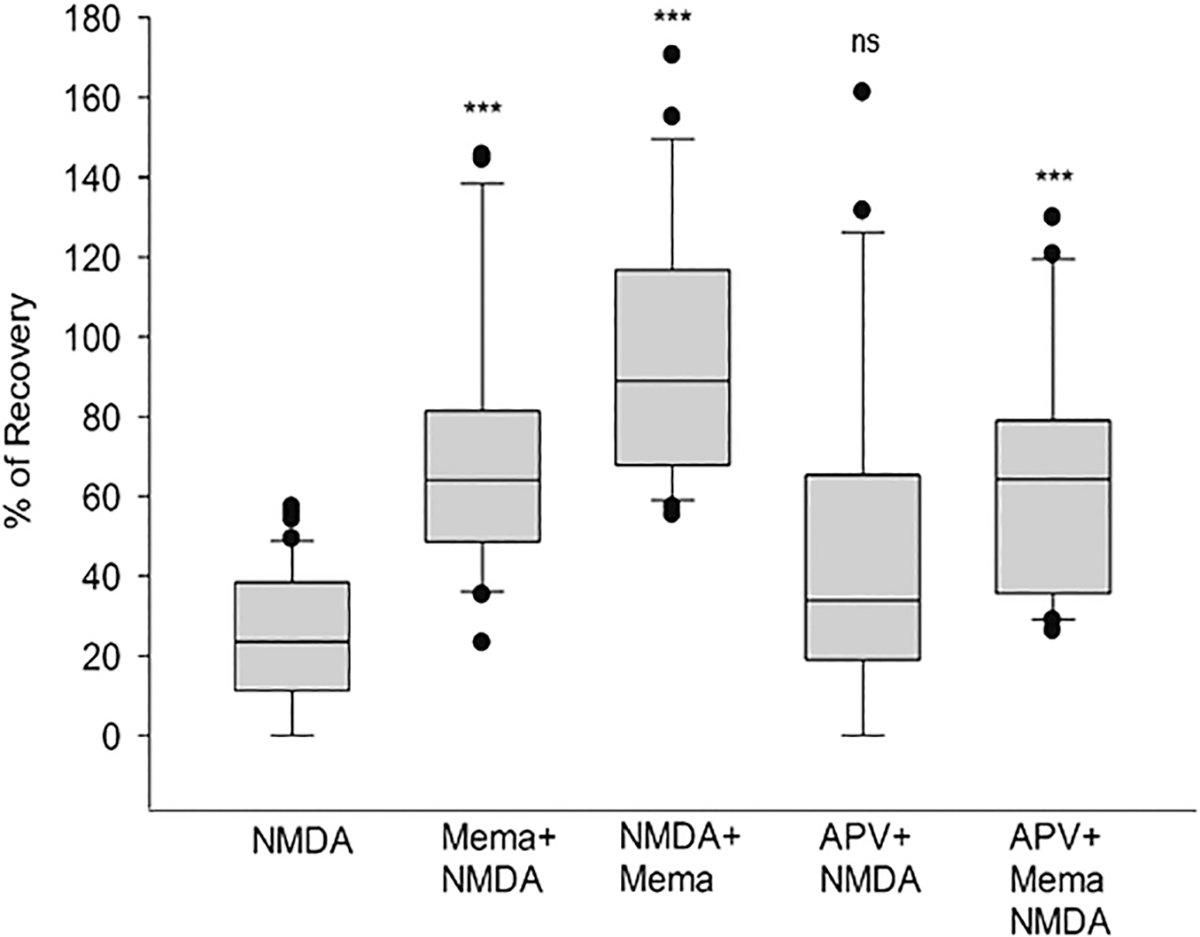
Memantine (Mema) can prevent and revert NMDA-induced excitotoxicity. Mema (1 μM) was superfused 15 min before (Mema+NMDA) or after (NMDA+Mema) the NMDA 0.5 mM insult to observe if it could prevent and revert NMDA-induced excitotoxicity. The requirement for NMDA receptors in the neuroprotection induced by memantine was also evaluated using the NMDA inhibitor APV (50 μM). APV was added to slices alone (APV + NMDA) for 1 h or co-incubated with memantine for 1 h (APV was added 15 min before memantine +Mema+NMDA) before the NMDA insult. The percent of PS recovery for all samples was measured after the NMDA insult (10 min) plus 1 h wash out (1:10 h after adding insult). One-way ANOVA: ***p < 0.001 in NMDA+Mema vs. NMDA; Mema+NMDA vs. NMDA; APV + Mema+NMDA vs. NMDA.

Next, we asked whether memantine could induce neuroprotection when applied before the NMDA insult to evaluate memantine’s potential as a pre-treatment or a preventive drug for excitotoxicity. In these experiments, 1 μM of memantine was superfused in hippocampal slices for 1 h before the NMDA insult (10 min), followed by a 1 h wash out. A significant PS recovery of 70% (mean value) (*p* < 0.001) was observed (Fig. 2, box 2). In a different experiment, memantine was superfused for 1 h after the 10 min NMDA insult, and a 95% recovery (p < 0.001) was observed (Fig. 2, box 3). Therefore, memantine 1 μM can prevent and revert NMDA excitotoxicity. Next, to test if the neuroprotective mechanism of memantine before NMDA insult was mediated by antagonism of this receptor, the NMDA receptor antagonist D(−)-2-Amino-5-phosphonopentanoic acid (APV) (50 μM, 100 times its K_i_.) (Ogden and Traynelis, 2011.) was superfused before NMDA insult with and without memantine co-incubation. When APV alone was applied for 1 h before the 10 min NMDA insult, then washed out for 1 h, the final PS showed no significant recovery (34% PS recovery, *p* = 0.082) (Fig. 2, box 4). When APV and memantine were co-applied for 1 h before the NMDA insult and then washed out, the final PS recovered up to 64% (*p* < 0.001) (Fig. 2, box 5). These experiments demonstrate that memantine uses an additional neuroprotective mechanism besides the NMDA-mediated uncompetitive antagonism since it induces neuroprotection even with a blockade of the NMDA receptor by APV. The box plots shown in Fig. 2 indicate the mean, standard deviation (SD), standard error of the mean (SEM), median (50 percentile), 25 percentile and 75 percentile of population spike recovery in the slices, and statistical analyses: NMDA alone results: Mean = 24.21%; SD = 17.44%; S.E.M = 2.69%; box plot: median = 23.57%; 25% = 11.19%; 75% = 38.41%, (*n* = 42). Mema+NMDA results: Mean = 69.99%; SD = 32.06%; S.E.M = 6.99%; box plot: median = 64.01%; 25% = 48.56%; 75% = 81.48%, (*n* = 21). NMDA+Mema results: Mean 94.64%; SD 31.05%; S.E.M. = 6.77%; box plot median = 88.93%; 25% = 67.84%; 75% = 116.75%, (n = 21). APV + NMDA results: Mean = 48.14%; SD = 42.76%; S.E.M = 9.31%; box plot: median = 33.90%; 25% = 18.92%; 75% = 65.29%, (n = 21). APV + Mema+NMDA results: Mean = 63.67%; SD = 31.61%; S.E.M = 6.90%; box plot: median = 64.28%; 25% = 35.61%; 75% = 79.06%, (n = 21). One-way ANOVA: ***p < 0.001 in NMDA+Mema vs. NMDA; Mema+NMDA vs. NMDA; Mema + NMDA vs. NMDA; APV + Mema + NMDA vs. NMDA; APV + NMDA vs. NMDA; and not significant (ns) p = 0.082, in APV + NMD.

### 3.3. Vesamicol inhibits the neuroprotective effect of 1 μM memantine

**Fig. 3.**
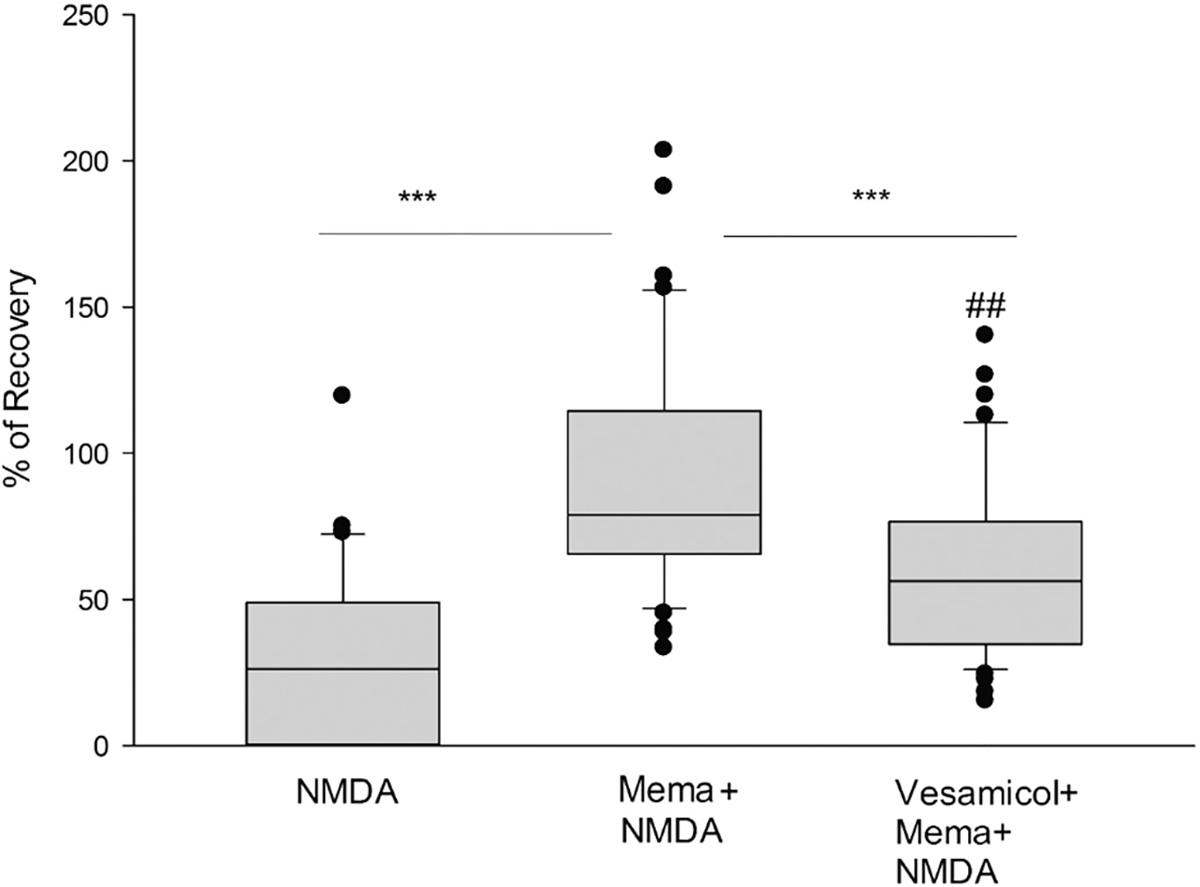
Vesamicol inhibits the neuroprotective effect of 1 μM memantine. To assess if memantine-induced neuroprotection requires acetylcholine release, vesamicol (50 μM), an inhibitor of vesicular acetylcholine transporter, was co-incubated with memantine (1 μM). Hippocampal slices were exposed to NMDA (0.5 mM, 10 min.) after incubation with memantine or vesamicol plus memantine for 1 h. Percent of PS recovery was measured one hour after NMDA insult and wash out for each condition. One-way ANOVA: ***p < 0.001, Mema+NMDA vs. NMDA and Vesamicol+Mema+NMDA vs. NMDA; ##p = 0.003 Mema+NMDA vs. Vesamicol+Mema+NMDA.

The following experiments aimed to dissect the alternative neuroprotective mechanisms of memantine pharmacologically (experimental methods shown in Supplementary Fig. 1). To determine whether memantine’s neuroprotection requires acetylcholine release, vesamicol, a non-competitive inhibitor of vesicular acetylcholine (ACh) transport (2-[4-phenylpiperidino] cyclohexanol), was tested in our system. Vesamicol inhibits the ACh transporter in the vesicles, thus decreasing the release of acetylcholine. In these experiments, memantine or memantine co-incubated with vesamicol for 1 h before the NMDA insult and wash out were evaluated. As in the previous results, slices incubated with memantine alone recovered their PS after the insult by 92% (*p* < 0.001) (Fig. 3, box 2). When vesamicol (50 μM) and memantine were co-applied before the insult for 1 h, the PS recovery after NMDA and wash out decreased to 60% (*p* < 0.001, Vesamicol+Mema+NMDA vs. NMDA), but it was not abolished (*p* < 0.003 Vesamicol+Mema+NMDA vs. Mema+NMDA) (Fig. 3, box 3). These results indicate that another mechanism, besides NMDA antagonism, contributes to memantine’s neuroprotective effect, which involves acetylcholine release. However, vesamicol decreased but did not abrogate memantine’s neuroprotection. This suggests that cholinergic vesicle release is part of the protective mechanism of memantine, but it is not the only route that mediates the beneficial effect of this drug in the brain at 1 μM. Details of the box plots shown in Fig. 3 indicating the percentile of population spike recovery in the slices, and statistical analyses: NMDA results: Mean = 30.13%; SD = 27.15%; S.E.M. = 4.19%; box plot: median = 26.25%; 25% = 0%; 75% = 48.96%, (*n* = 42); Mema+NMDA Mean = 91.66%; SD = 40.94%; S.E. M. = 6.32%; median 78.89%; 25% = 65.50%; 75% = 114.57%, (n = 42). Vesamicol+Mema+NMDA results: Mean = 60%; SD = 29.98%; S.E.M. = 4.63%; box plot: median = 56.35%; 25% = 34.66%; 75% = 76.50%, (n = 42). One-way ANOVA: ***p < 0.001, Mema+NMDA vs. NMDA and Vesamicol + Mema + NMDA vs. NMDA; ##*p* = 0.003 Mema+NMDA vs Vesamicol+Mema+NMDA.

### 3.4. Nifedipine inhibits the neuroprotective effect of 1 μM memantine

**Fig. 4.**
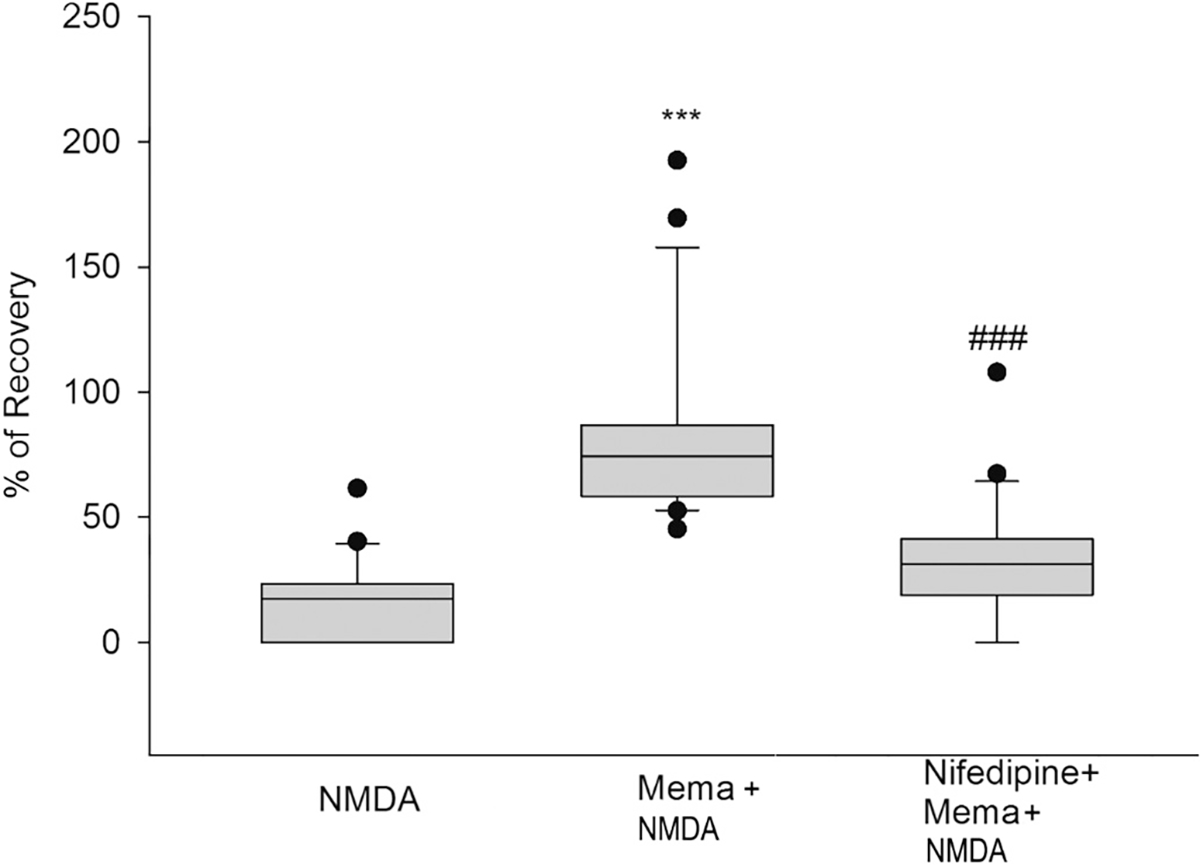
Nifedipine inhibits the neuroprotective effect of 1 μM memantine. Hippocampal slices were exposed to NMDA (0.5 mM, 10 min) after incubation with memantine (1 μM) or with memantine plus the L-type calcium channel blocker, nifedipine (10 μM), for 1 h. Percent of PS recovery was measured one hour after NMDA insult and wash out for each condition. One-way ANOVA: ***p < 0.001 when Nifedipine+Mema+NMDA is compared to NMDA, ###p < 0.001 when Nifedipine+Mema+NMDA is compared to Mema+NMDA.

According to the literature, inhibiting L-type Ca^2+^ channels is a potential therapeutic target for AD that involves vasodilation and protection from vascular injury (Anekonda et al., 2011). To evaluate the pathway of neuroprotection by memantine, blockade of L-type Ca^2+^ channels was tested in our system using the drug nifedipine. The application of memantine alone for 1 h before the NMDA insult led to subsequent PS recovery of 82% (*p* < 0.001, Mema+NMDA vs. NMDA) (Fig. 4, box 2). When nifedipine (10 μM) was co-administered with memantine, neuroprotection of memantine after the NMDA insult was abolished, decreasing PS recovery to 31% (p < 0.001, Nife+Mema+NMDA vs. Mema+NMDA) (Fig. 4, box 3). Thus, L-type Ca^2+^ channel activity is essential for memantine’s neuroprotective effect against NMDA-induced neurotoxicity. These results demonstrate that the therapeutic effect of memantine at 1 μM requires the activity of the L-type Ca^2+^channels. Details of the box plots shown in Fig. 4 indicating the percentile of population spike recovery in the slices, and statistical analyses: NMDA results: Mean = 16.11%; SD = 17.22%; S.E.M. = 3.76%; box plot: median = 17.41%; 25% = 0%; 75% = 23.46%, (*n* = 21). Mema+NMDA results: Mean = 82.26%; SD = 36.64%; S.E.M. = 7.80%; box plot: median = 74.36%; 25% = 58.34%; 75% = 86.89%, (n = 21). Nifedipine+Mema+NMDA results: Mean = 31.77%; SD = 25%; S.E.M. = 5.46%; box plot: median = 31.19%; 25% = 18.90%; 75% = 41.29%, (n = 21). One-way ANOVA: *** *p* < 0.001 when Nifedipine+Mema+NMDA is compared to NMDA, ### *p* < 0.001 when Nifedipine+Mema+NMDA is compared to Mema+NMDA.

### 3.5. 4R and MLA do not use L-type calcium channels to induce neuroprotection

**Fig. 5.**
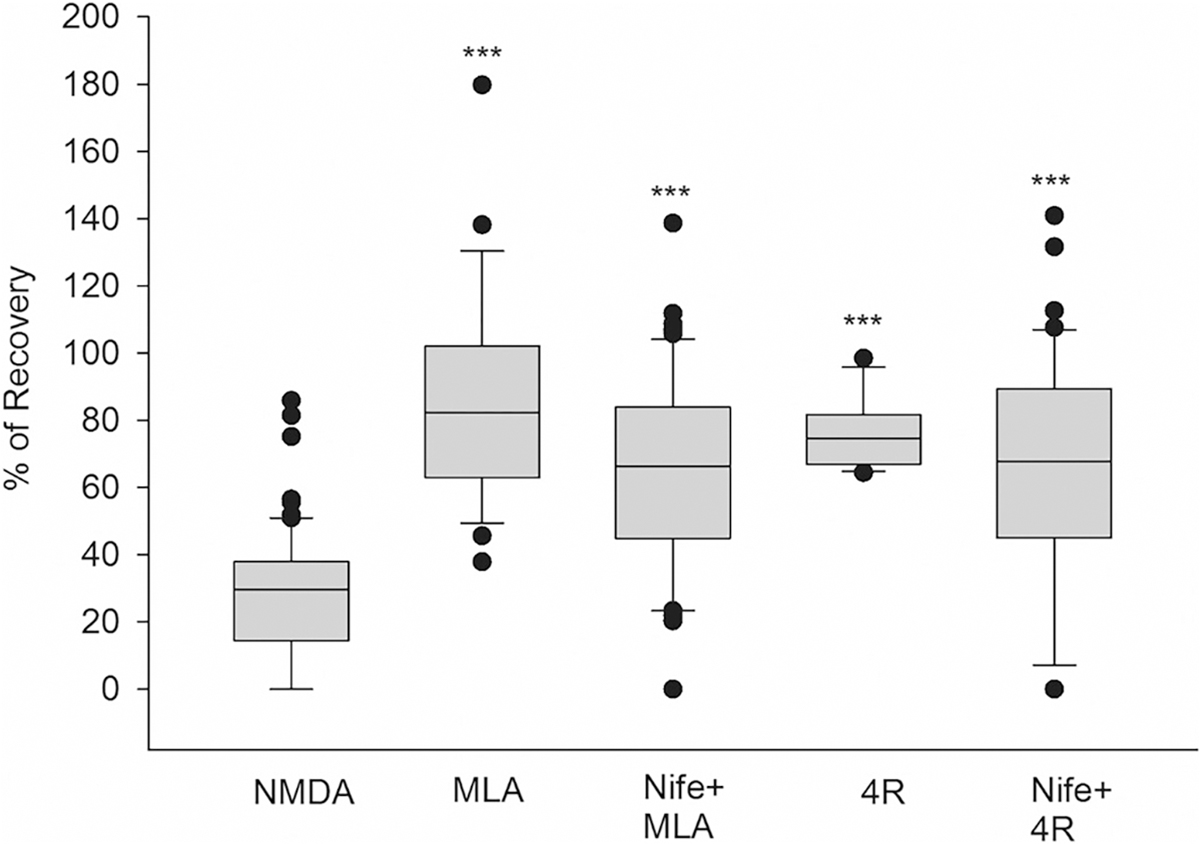
The 4R cembranoid and MLA do not use L-type calcium channels to induce neuroprotection. Hippocampal slices were exposed to NMDA (0.5 mM, 10 min) after 1 h incubation with MLA (10 nM) and MLA plus the L-type calcium channel blocker, nifedipine (Nife, 10 μM). The same incubation protocol was conducted using 4R (2 μM) and 4R plus Nife (10 μM). Percent of PS recovery was measured one hour after NMDA insult and wash out for each condition. One-way ANOVA: for all groups, ***p < 0.001 compared to NMDA control (Dunn’s test). There is no significant difference between nifedipine treatments, MLA and 4R.

Our previous experiment showed that memantine uses nicotinic pathways for its neuroprotective effects. Other drugs that induce neuroprotection against NMDA excitotoxicity mediated by nicotinic pathways are the tobacco 4R cembranoid and the α7 nAChR inhibitor methyllycaconitine (MLA). The following experiments tested whether 4R and MLA use neuroprotection pathways similar to memantine (Fig. 5). Nifedipine was applied to test if 4R and MLA’s neuroprotection involves the use of L-type Ca^2+^ channels. MLA (10 nM) was added to the slices for 1 h, NMDA insult was conducted for 10 min, and slices were washed for 1 h. PS after MLA treatment had a recovery of 85% (Fig. 5, box 2) (***p < 0.001) compared to NMDA, showing its expected neuroprotective effect. In these experiments, nifedipine (10 μM) was co-incubated with MLA (10 nM) for 1 h before the NMDA insult for 10 min, followed by a 1 h wash-out. The resulting PS recovery decreased to 64% (Fig. 5, box 3) (***p < 0.001 for Nife+MLA vs. NMDA). In the case of 4R (2 μM), the same protocol was followed. When 4R alone was superfused one hour before the NMDA insult, the PS recovery was 76% (Fig. 5, box 4) (***p < 0.001) compared to NMDA alone. When nifedipine was co-incubated with 4R for 1 h, PS recovered at 64% (Fig. 5, box 5) (***p < 0.001) compared to NMDA. In general, 4R and MLA alone generated a statistically significant PS recovery compared to NMDA alone (Fig. 5, boxes 1, 2, 4). No statistical significance was found between 4R and MLA treatments alone in PS recovery (Fig. 5, boxes 2, 4). To our surprise, no difference was found in the PS recovery of either drug alone or co-incubated with nifedipine (Fig. 5, boxes 2–5). These results suggest that 4R could mediate its neuroprotective effect through similar mechanisms to MLA, and interestingly, it does not require the activity of L-type Ca + 2 channels. Details of the box plots shown in Fig. 5 indicating the percentile of population spike recovery in the slices, and statistical analyses: NMDA results: Mean = 27.99%; SD = 19.51%; S. E.M. = 2.33%; box plot: median = 29.69%; 25% = 14.49%; 75% = 37.88%, (*n* = 70). MLA + NMDA results: Mean = 84.95%; SD = 30.91%; S.E.M. = 5.84%; box plot: median = 82.30%; 25% = 62.95%; 75% = 102.08%, (*n* = 28). Nife+MLA + NMDA results: Mean = 63.89%; SD = 29.12%; S.E.M. = 3.67%; box plot: median = 63.32%; 25% = 44.82%; 75% = 84.05%, (n = 63). 4R + NMDA results: Mean 76.48%; SD = 10.28%; S.E.M. = 2.75%; box plot: median = 74.47%; 25% = 66.87%; 75% = 81.60%, (*n* = 14). Nife+4R + NMDA results: Mean = 64.01%; SD = 34.24%; S.E.M. = 5.28%; box plot: median = 67.75%; 25% = 44.92%; 75% = 89.36%, (*n* = 42). One-way ANOVA: for all groups, *p* < 0.001*** compared to NMDA control (Dunn’s test). There is no significant difference between nifedipine treatments, MLA, and 4R.

### 3.6. Memantine requires α4β2 to induce neuroprotection

**Fig. 6.**
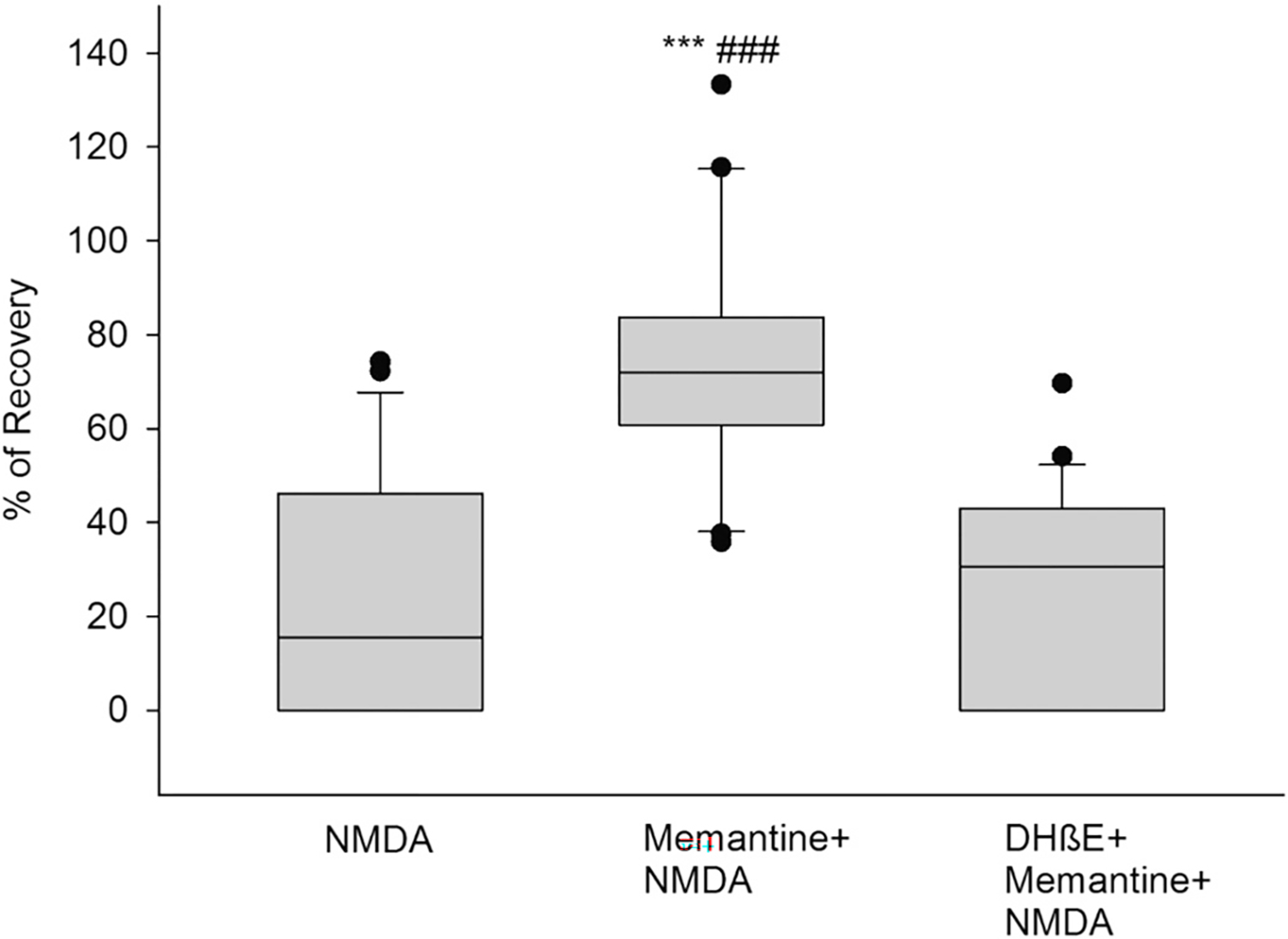
The α4β2 nAChR inhibition abolishes memantine’s neuroprotection against NMDA. Hippocampal slices were exposed to NMDA (0.5 mM, 10 min) after incubation with memantine (1 μM) or memantine plus the α4β2 inhibitor DHβE (1 μM) for 1 h. Percent of PS recovery was measured one hour after NMDA insult and wash out for each condition. One-way ANOVA: ***p < 0.001, Mema+NMDA vs. NMDA, ###p < 0.001 Mema+NMDA vs. DHβE+Mema+NMDA.

Considering that memantine uses acetylcholine release in part to induce its neuroprotective effect, we tested if it required the abundant α4β2 nicotinic acetylcholine receptor activity. To achieve this, a 1 uM Dihydro-β-erythroidine (DHβE) (Raggenbass and Bertrand, 2002), a selective inhibitor of the α4β2 receptor, was used. For controls, memantine alone was superfused for 1 h before NMDA insult (*p* < 0.001, Mema+NMDA vs. NMDA) (Fig. 6, box 2). In experimental samples, when memantine was co-administered with DHβE for 1 h, the PS recovery after NMDA insult and wash out was 24% (p < 0.001, Memantine +NMDA vs. DHβE+Mema+NMDA), very similar to the NMDA alone PS recovery of 25% (Fig. 6, boxes 1 and 3). Therefore, these results demonstrate that memantine’s neuroprotection against NMDA-induced neurotoxicity depends on the cholinergic activity of the α4β2 nAChR. Details of the box plots shown in Fig. 6 indicate the percentile of PS recovery in the slices, and statistical analyses: NMDA results: Mea*n* = 21.81%; SD = 24.51%; S.E.M. = 5.35%; box plot: median = 15.54%; 25% = 0%; 75% = 46.11%, (n = 21). Mema+NMDA results: Mean = 74.56%; SD = 25.22%; S.E.M. = 5.50%; box plot: median = 72.03%; 25% = 60.79%; 75% = 83.73%, (*n* = 21). DHβE+Mema+NMDA results: Mean = 23.90%; SD = 22.07%; S.E.M. = 4.82%; box plot: median = 30.52%; 25% = 0%; 75% = 42.93%, (n = 21). One-way ANOVA: ***p < 0.001, Mema+NMDA vs. NMDA, ###p < 0.001 Mema+NMDA vs. DHβE+Mema+NMDA.

### 3.7. The neuroprotection elicited by memantine is not dependent on the MAPK/ERK pathway

**Fig. 7.**
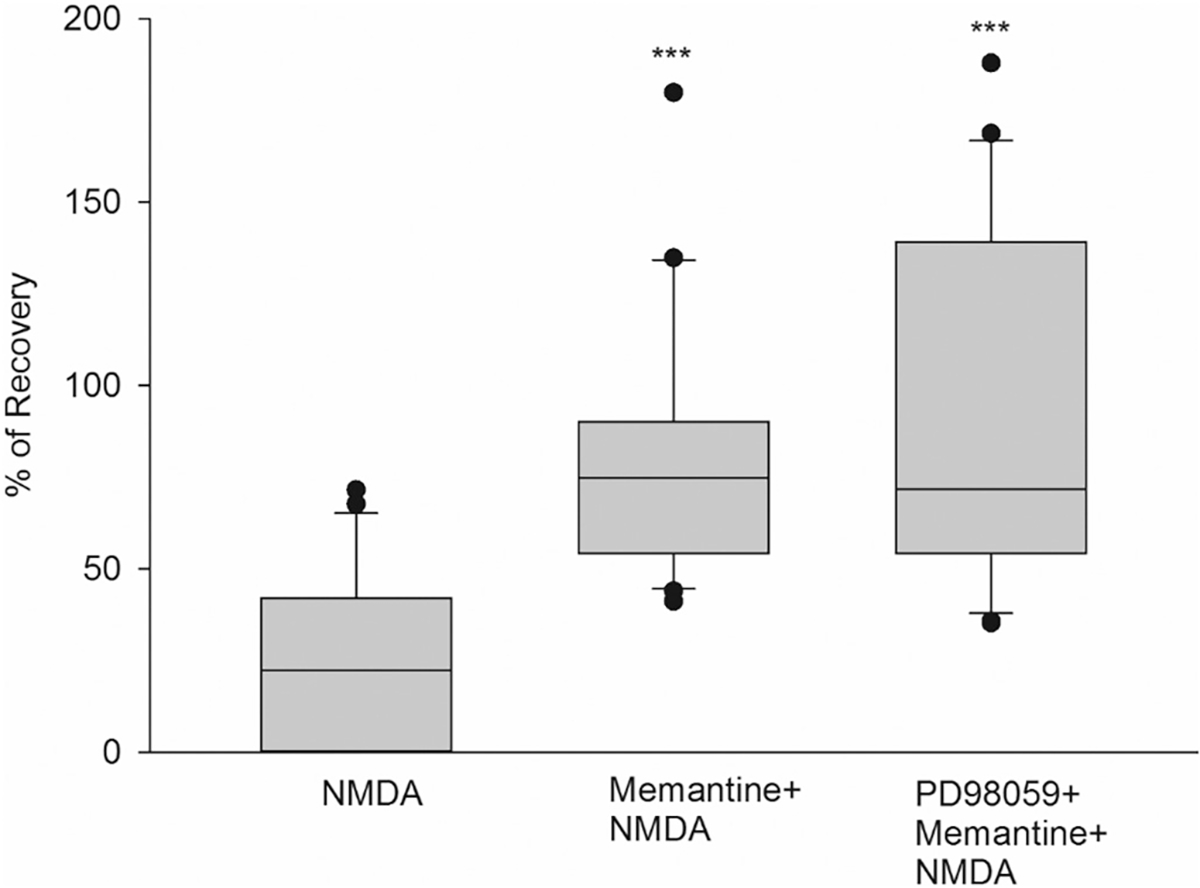
The neuroprotection elicited by memantine is not dependent on the MAPK/ERK pathway. Hippocampal slices were exposed to NMDA (0.5 mM, 10 min) after incubation with memantine (1 μM) and memantine plus the MAPK/ERK inhibitor PD98059 (50 μM) for 1 h. Percent of PS recovery was measured one hour after NMDA insult and wash out for each condition. One-way ANOVA: ***p < 0.001, Mema+NMDA vs. NMDA, ***p < 0.001, PD98059 + Mema+NMDA vs. NMDA; and not significant, p = 0.945 in Mema+NMDA vs. PD98059 + Mema+NMDA.

Neuroprotection against NMDA-induced excitotoxicity can occur by activating PI3K or the MAPK/ERK signaling pathway, among other mechanisms(Yu and Koh, 2017; Kheiri et al., 2018). To determine whether memantine uses any of these two mechanisms, we asked whether blocking the MAPK/ERK or PI3K signaling pathways could inhibit the neuroprotective effects of memantine (Fig. 7). First, the MAPK/ERK signaling cascade was tested using PD98059, a MAPK/ERK pathway inhibitor. In these experiments, hippocampal slices were superfused with memantine or memantine plus PD98059 for 1 h, exposed to the NMDA insult (10 min), and wash out for 1 h. With memantine alone, PS recovered 78% compared to NMDA alone (Fig. 7, box 2) (***p < 0.001). Co-incubation of memantine with PD98059 (50 μM) showed a PS recovery of 89% (***p < 0.001) (Fig. 7, box 3) compared to NMDA. There was no significant recovery difference between Mema+NMDA and PD98059 + Mema+NMDA (Fig. 7, boxes 2 and 3) (*p* = 0.945). These results show that MAPK/ERK inhibition did not interfere with memantine’s neuroprotective effect, demonstrating that memantine does not employ the MAPK/ERK pathway to protect from NMDA excitotoxicity. Details of the box plots shown in Fig. 7 indicate the percentile of population spike recovery in the slices, and statistical analyses: NMDA results: Mean = 24.27%; SD = 22.74%; S.E. M. = 4.96%; box plot: median = 22.38%; 25% = 0%; 75% = 42.02%, (n = 21). Mema+NMDA results: Mean = 78.29%; SD = 34.60%; S.E.M. = 7.55%; box plot: median = 74.77%; 25% = 54.07%; 75% = 90.12%, (n = 21). PD98059 + Mema+NMDA results: Mean = 89.21%; SD = 47.13%; S.E.M. = 10.28%; box plot: median = 71.75%; 25% = 54.14%; 75% = 139.25%, (n = 21). One-way ANOVA: ***p < 0.001, Mema+NMDA vs NMDA, ***p < 0.001, PD98059 + Mema+NMDA vs NMDA; and not significant, p = 0.945 in Mema+NMDA vs. PD98059 + Mema+NMDA.

### 3.8. The mechanism of memantine to induce neuroprotection is partly mediated by the PI3K pathway

**Fig. 8.**
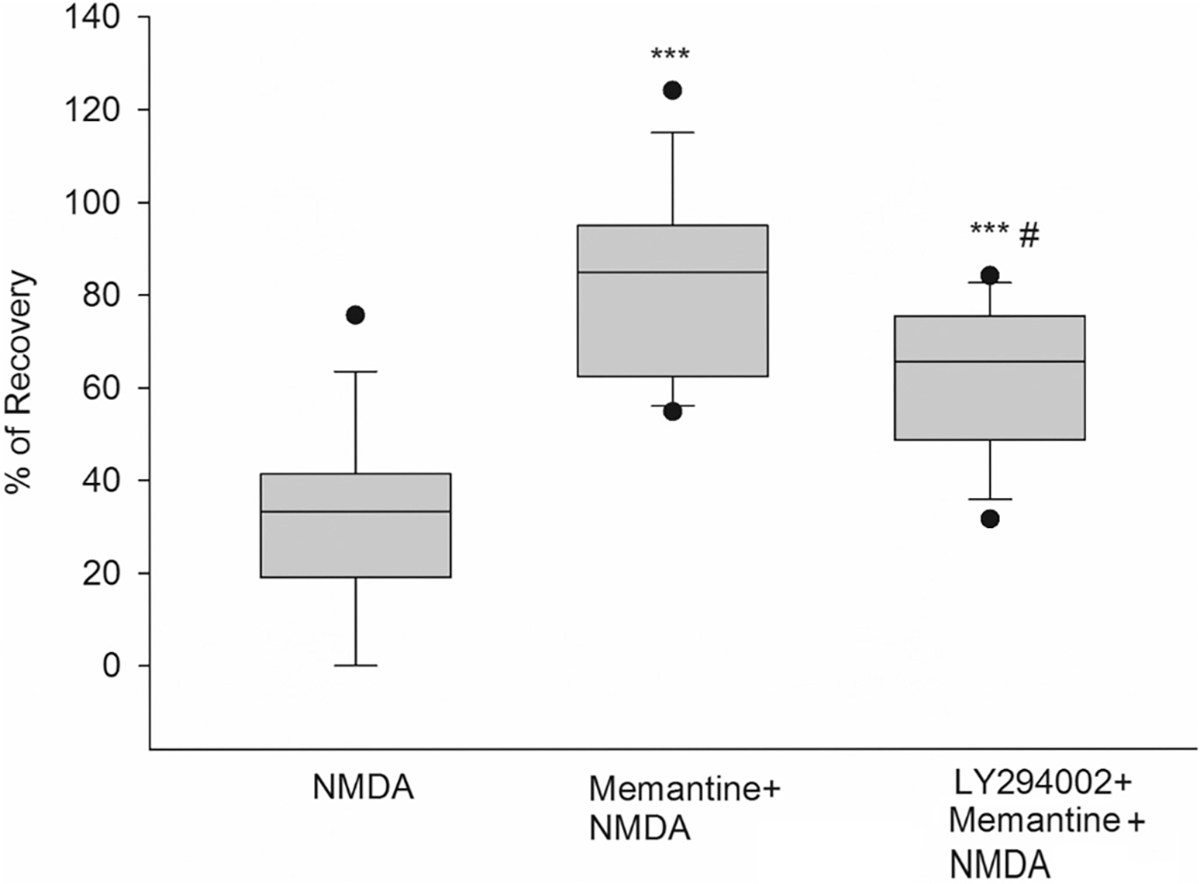
Memantine-mediated neuroprotection is partially attributed to PI3K activity. Hippocampal slices were exposed to NMDA (0.5 mM, 10 min) after incubation with memantine (1 μM) and memantine plus the PI3K inhibitor LY294002 (50 μM) for 1 h. Percent of PS recovery was measured one hour after NMDA insult and wash out for each condition. One-way ANOVA: ***p < 0.001 LY294002 + Mema+NMDA is compared to NMDA, #p < 0.01 when LY294002 + Mema+NMDA is compared to Mema+NMDA. NMDA vs. Mema+NMDA is ***p < 0.001.

To test whether the phosphoinositide 3-kinase (PI3K)/AKT pathway mediates memantine neuroprotection, a selective inhibitor of PI3K, LY294002, was used. Following the same methodology as the previous assays, memantine was superfused for 1 h and after the addition and wash out of NMDA, PS recovered by 85% (Fig. 8, box 2) (***p < 0.001) compared to NMDA alone. When LY294002 (10 μM) was co-incubated with memantine for 1 h, PS recovery after NMDA was reduced to 62% (*p < 0.01 for LY294002 + Mema+NMDA vs. Mema+NMDA) (Fig. 8, box 3). In this case, PS recovery decreased by 23% compared to memantine alone, while significantly maintaining a neuroprotective effect. These results indicate that PI3K is not essential, but is partially responsible, for memantine’s neuroprotection against NMDA-induced excitotoxicity. Details of the box plots shown on Fig. 8 indicate the percentile of population spike recovery in the slices, and statistical analyses: NMDA results: Mean = 31.51%; SD = 19.66%; S.E.M. = 5.25%; box plot: median = 33.25%; 25% = 19.10%; 75% = 41.35%, (*n* = 14). Mema+NMDA results: Mean 82.43%; SD 20.53%; S.E.M. = 5.49%; box plot: median = 84.83%; 25% = 62.34%; 75% = 94.99% (n = 14). LY294002 + Mema+NMDA results: Mean = 62.25%; SD = 16.23%; S.E. M. = 4.34%; box plot: median = 65.55%; 25% = 48.67%; 75% = 75.41%, (n = 14). One-way ANOVA: ***p < 0.001 LY294002 + Mema+NMDA is compared to NMDA, #p < 0.01 when LY294002 + Mema+NMDA is compared to Mema+NMDA. NMDA vs. Mema+NMDA is ***p < 0.001.

### 3.9. Neuroprotection against NMDA toxicity by tobacco cembranoids

**Fig. 9.**
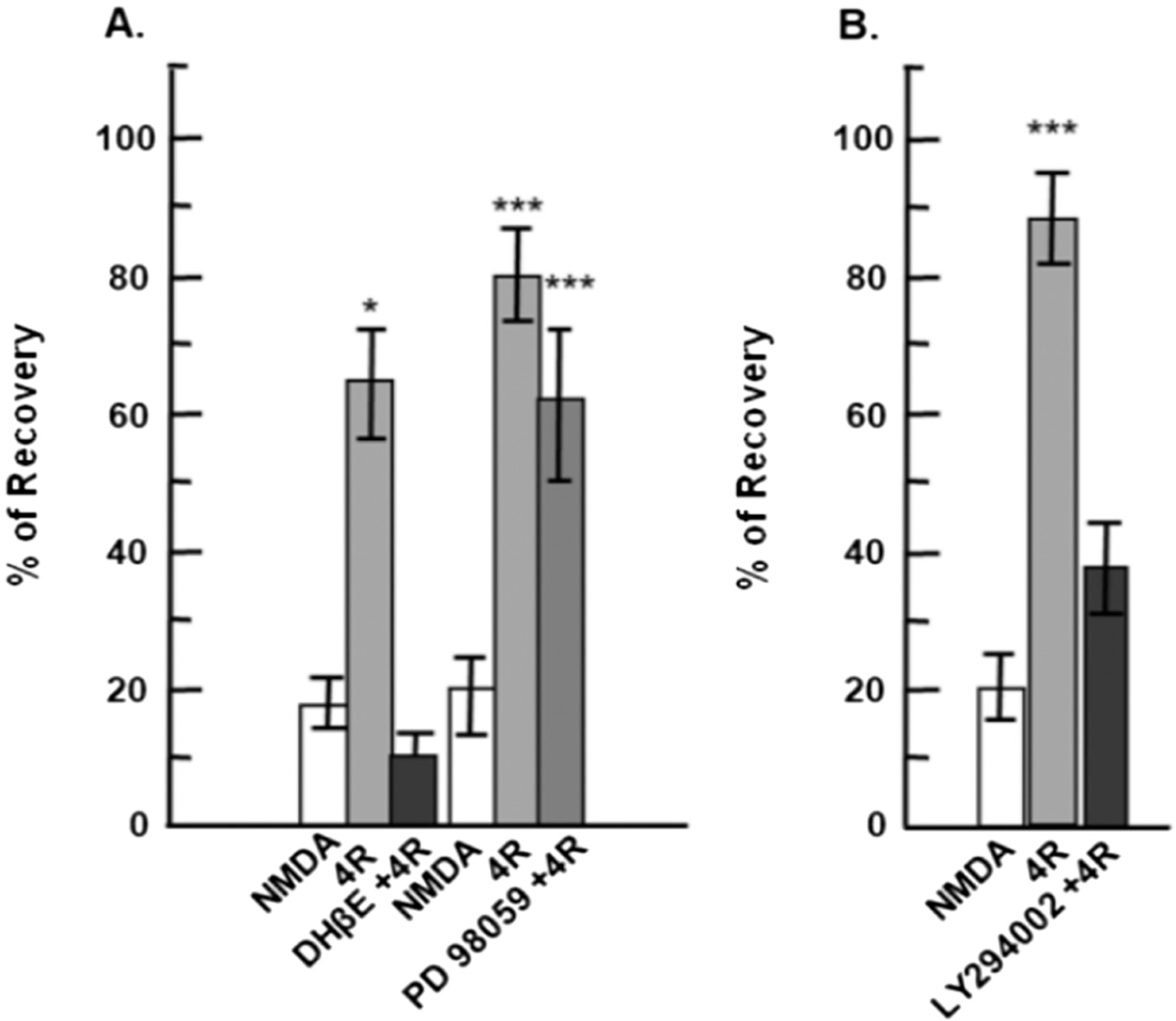
Molecular requirements of neuroprotection against NMDA toxicity by the 4R cembranoid. A. White columns represent the percent recovery of hippocampal slices exposed to 0.5 mM NMDA 10 min and 1 h wash out. This condition, referred to as NMDA control, shows the effect of NMDA toxicity in unprotected slices. The gray bars represent the PS recovery when slices were pre-incubated before the NMDA insult with additional treatments. Black and dark gray columns show neuroprotection when co-incubating 2 μM 4R with 1 μM DHβE or PD98059, respectively. In this figure, NMDA vs. 4R: *p < 0.05; *n* = 21 slices per group, NMDA vs. DHβE +4R is n.s.; n = 21 slices, and NMDA vs. PD98059 + 4R ***p < 0.002; n = 21 slices. B. Under the same conditions as A, 4R (gray column) mediated significant neuroprotection (NMDA vs. 4R ***p < 0.001; *n* = 20) compared to NMDA alone (white column), and LY294002 + 4R treated slices (black column) did not show a significant difference in recovery from NMDA controls (NMDA vs. LY294002 + 4R is n.s.; n = 21 slices). *Reprint from “Tobacco Cembranoids Protect the function of Acute Hippocampal SlicesAgainst NMDA by a Mechanism Mediated by α4β2 Nicotinic Receptors” by Ferchmin et al., 2005, Journal of Neuroscience Research 82:631–641. Modified and reproduced, with permission, from John Wiley and Sons license # 5184820641580.

The molecular cascades enabling 4R neuroprotection from an NMDA insult were investigated pharmacologically. The nicotinic receptor α4β2, MEK-1,2, kinases or PI3K/AKT phosphorylation, were examined using their respective inhibitors and antagonists: DHβE (1 μM), PD98059 (50 μM) and LY294002 (10 μM) (Fig. 9 A, B). In Fig. 9A, DHβE (1 μM) or the MEK-1,2 inhibitor PD98059 (50 μM) were superfused alone for 15 min, followed by a 1 h co-application with 2 μM 4R. After the 10 min NMDA insult and 1 h wash out, the percent of PS recovery was measured for each condition. The neuroprotection provided by 4R was inhibited by DHβE but not by the MEK-1,2 inhibitor PD98059. In Fig. 9B, 4R neuroprotection was inhibited by the PI3K inhibitor LY294002. These results show that both PI3K/AKT phosphorylation and α4β2 receptors, mediate 4R’s neuroprotective effect, but not MEK-1,2 (for details, see Ferchmin et al., 2005). Fig. 9 was modified and reproduced with permission from John Wiley and Sons license # 5184820641580.

## 4. Discussion

In 2003, Memantine was approved to treat moderate to severe AD in the USA as an NMDA receptor non-competitive antagonist of low affinity (Lo and Grossberg, 2011; Robinson and Keating, 2006; Rogawski and Wenk, 2003). This drug has shown no activity for the dopamine, gamma-aminobutyric acid (GABA), histamine, glycine, benzodiazepine, adrenergic receptors, or the voltage-dependent potassium, calcium, or sodium channels (Johnson and Kotermanski, 2006; Rammes et al., 2008). In addition, memantine shows antagonistic activity to the serotonergic type 3 (5-HT3) and the nicotinic acetylcholine receptors (Rogawski and Wenk, 2003). Although the most recognized mechanism of action for memantine is the non-competitive antagonism of the NMDA receptor (Lipton, 2006; Johnson and Kotermanski, 2006; Song et al., 2018), other publications recognize its potential to inhibit the α7 nAChR as a pharmacological target for neurological diseases (Maskell et al., 2003). The comprehensive review of (Folch et al., 2018) presents the therapeutic potential of memantine in the context of AD disease. Although in preclinical studies, memantine was successful, in clinical trials, memantine was less effective. Our data suggest that memantine is neuroprotective when it targets α7 nACh-R at concentrations below 3 μM. At higher concentrations, memantine inhibits the neuroprotective NMDA-R and becomes neurotoxic (see Fig. 1). In agreement with our in vitro data, similar concentration dependence of memantine was observed in vivo by Trotman (Trotman et al., 2015). Our work shows a strong correlation between memantine’s capacity as a neuroprotective agent and its dependency on nicotinic receptors and signaling. This study also shows that the neuroprotective potential of memantine goes beyond NMDA inhibition alone since memantine protects before an excitotoxic NMDA insult by 70%, and after the insult, by 95%. To test if blockade of the NMDA receptor accounted for memantine’s neuroprotection, the NMDA antagonist APV was applied. Co-administration of APV (IC_50_ = 0.5 μM) with memantine did not affect memantine’s neuroprotection when added before an NMDA insult, showing a 64% recovery (APV + Mema+NMDA), similar to Mema+NMDA alone. When memantine is not present, APV + NMDA’s neuroprotection decreases to 48%. APV has a greater affinity than memantine to the NMDA receptor (Ogden and Traynelis, 2011). It is counter-intuitive that inhibition of the NMDA receptor could decrease the neuroprotection against NMDA-induced excitotoxicity. However, the FDA approved the NMDA antagonist memantine because of its exceptional kinetic properties that produce a mild non-neurotoxic inhibition of the NMDA receptor (Lipton, 2006), distinct from APV. Overstimulation of the NMDA receptor is known to be the cause of excitotoxic neuronal death or injury. Therefore, the inhibition of the NMDA receptor was assumed to be the solution for excitotoxic and neurodegenerative diseases. A plethora of NMDA receptor antagonists were tested and proved to be neurotoxic because the activity of NMDA receptors is vital for neuronal survival. After a traumatic excitotoxic episode, the NMDA receptors assume their normal physiological functions, which include promoting neuronal survival (Hardingham et al., 2002; Soriano et al., 2006; Papadia et al., 2008). The NMDA receptor antagonists failed in the clinical trials of stroke and traumatic brain injury because the blockade of synaptic transmission mediated by NMDA receptors is neurotoxic (Ikonomidou and Turski, 2002; Papadia et al., 2008).

To test the nicotinic component of memantine’s neuroprotective mechanism, we blocked ACh uptake into synaptic vesicles using vesamicol (Kaufman et al., 1989; Prior et al., 1992). The neuroprotection induced by memantine was significantly decreased by vesamicol, indicating that acetylcholine release with posterior activation of nicotinic receptors is necessary to induce neuroprotection. Interestingly, this is one of the common mechanisms of neuroprotection between 4R, methylcaconitine (MLA), and memantine since 4R and MLA neuroprotection from NMDA were shown to be blocked by vesamicol in previous studies (Ferchmin et al., 2005, 2013). Another common mechanism of neuroprotection shared by 4R, MLA (Ferchmin et al., 2005), and memantine, reported in these studies, is their dependency on the activity of the α4β2 nAChR. This was demonstrated by their inhibition of neuroprotection after the addition of the α4β2 nAChR inhibitor DHβE in hippocampal slices. Based on the K_i_ values for different neuronal nicotinic receptors, 1 μM DHβE inhibits α4β2 with high selectivity (Eaton et al., 2003; Sharples and Wonnacott, 2001). Thus, we ensured that the dose used in our experiments of 1 μM was adequate to block these receptors without interfering with other receptors. Other studies have also confirmed the neuroprotection-mediating role and therapeutic potential of the nicotinic α4β2 receptor in insults such as Parkinson’s disease, showing its relevance in different conditions (Quik and Wonnacott, 2011; Quik et al., 2013).

Additionally, we found that blocking L-type VGCC using nifedipine decreased memantine neuroprotection, and other researchers have found that calcium signaling mediated by L-type calcium channels was necessary to induce neuroprotection (Dolmetsch et al., 2001). Interestingly, nifedipine did not interfere with the neuroprotection triggered by 4R or MLA, which is inconsistent with (Ferchmin et al., 2005), which was reproduced several times. Although activation of L-type calcium channels is still part of nicotinic neuroprotection in drugs such as memantine, alternative L-type calcium channel-independent nicotinic mechanisms can still be neuroprotective through different pathways. These drugs could be protective, in part, by not exacerbating calcium dysregulation after an excitotoxic insult (Arundine et al., 2003). Some groups are testing L- and V-type calcium channel blockers to protect against the calcium excitotoxicity induced by neurotoxins such as organophosphates (Fernandes et al., 2017). Taken together, the mechanisms of neuroprotective drugs that do not require the activation of L-type calcium channels, such as 4R, could provide therapeutic benefits. These drugs could have a wider dosage range since they do not contribute to calcium dysregulation during excitotoxicity.

Our studies of nicotinic downstream pathways demonstrated that memantine, just like 4R’s and MLA’s neuroprotection against NMDA, is not dependent on the MAPK / ERK kinase pathway (RAF/MEK/ERK pathway). In our experiments, 50 μM of the MAPK/ERK inhibitor PD98059 did not reduce the neuroprotection by any tested drug. These results agree with previous studies of nicotinic inhibition mechanisms carried out by Aracava et al., 2005 and Ferchmin et al., 2003, 2014. When we continue comparing the neuroprotective mechanisms of action of nicotinic inhibitors, we found that both 4R and memantine require AKT phosphorylation in serine 473 to induce neuroprotection, although in different amounts. In the case of 4R, its neuroprotection was significantly inhibited by LY294002, decreasing the PS from 90% to 40%. These results suggest that 4R’s main pathway is the anti-apoptotic, pro-survival PI3K/AKT pathway after an NMDA-excitotoxicity insult. Our findings showed that memantine’s neuroprotection after LY294002 significantly decreased by 20% (*p* < 0.01). Although this neuroprotective pathway might not be the primary mechanism for memantine, other studies have confirmed the use of the PI3K/AKT pathway by this drug, which confers anti-apoptotic activity (Jantas and Lasoń, 2009; Feng et al., 2020).

To understand the nicotinic mechanisms of hippocampal neuroprotection induced in our studies, we must consider the cell types mediating nicotinic signaling in this brain region. Nicotinic receptors are expressed on interneurons, and incoming presynaptic fibers, which are known to contain nAChRs (Kawai et al., 2002; Liu et al., 2012; McKay et al., 2007; Cheng and Yakel, 2015). It is conceivable that a small number of nAChRs are present in pyramidal neurons, as shown by functional α7 nAChR-mediated currents reported in CA3 and CA1 pyramidal neurons by Ji et al., 2001; Grybko et al., 2011; Gu et al., 2012, although several publications demonstrate the opposite (L. Khiroug et al., 2003; S. S. Khiroug et al., 2004).

The non-α7 and the α7 nACh receptors in glutamatergic and GABAergic synaptic terminals play an important role in mediating nicotinic effects on neurons in many brain regions (Alkondon and Albuquerque, 2002; Gray et al., 1996; Grillner et al., 2000; L. Khiroug et al., 2003). In the case of 4R, the NP is initiated by a decrease in the GABAergic tone (Ferchmin et al., 2005; Ferchmin et al., 2013). In short, 4R negatively modulates α7 nAChR on GABAergic interneurons or septal afferents, and decreased α7 activity reduces GABA release. Reduced inhibition on cholinergic terminals allows for more ACh release. This enhanced ACh release activates the α4β2 nACh receptors, triggering the PI3K/AKT neuroprotective cascade. The ACh-vesicle release inhibitor, vesamicol, and the α4β2 inhibitor DHβE block this neuroprotective pathway induced by 4R, and our study demonstrates the same results with memantine, suggesting similar neuroprotective pathways for these two nicotinic inhibitors.

**Fig. 10.**
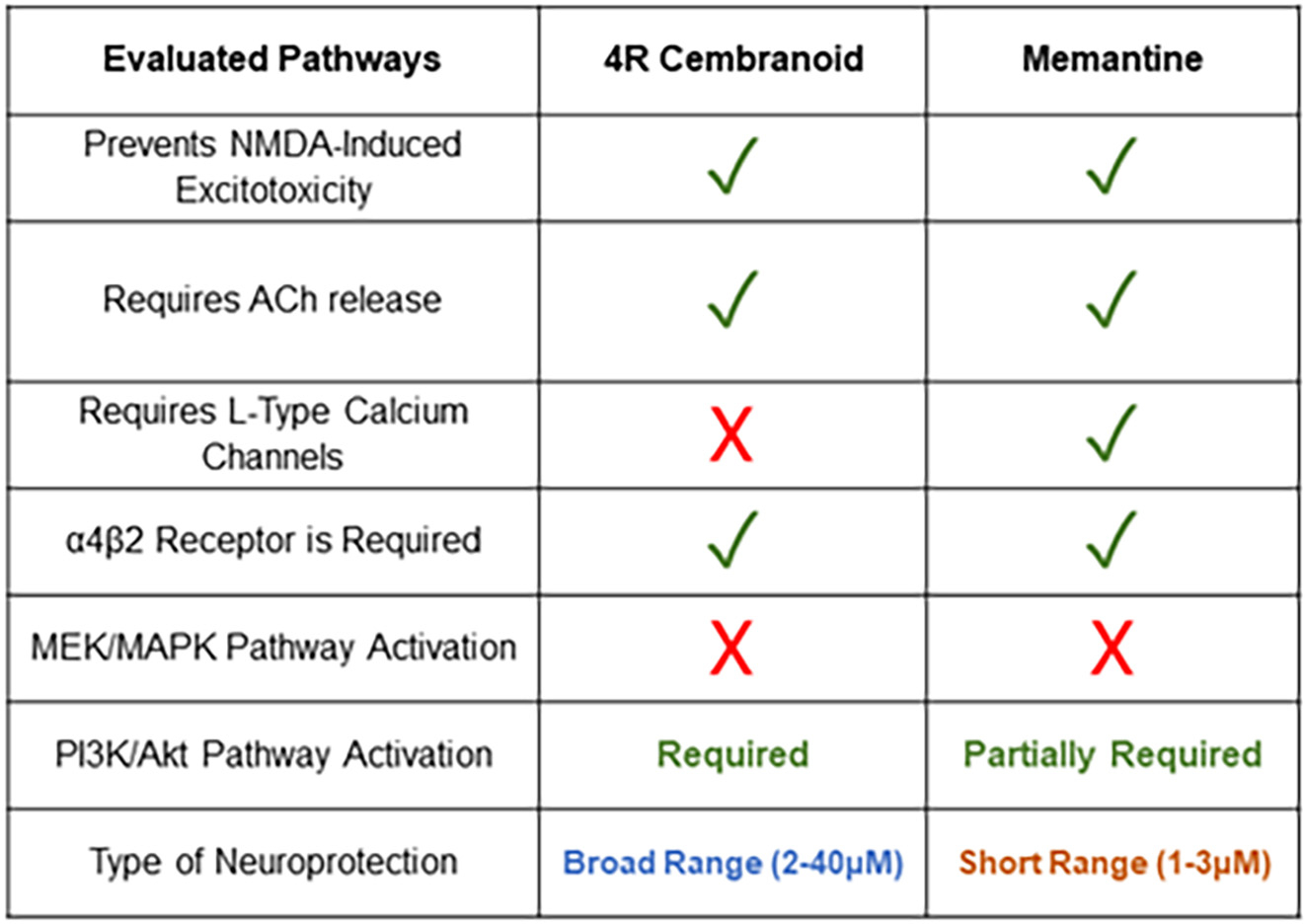
Summary of the evaluated mechanisms to compare 4R and memantine’s cholinergic neuroprotection. A comparison of the cholinergic mechanisms leading to neuroprotection between 4R and memantine shows that five were similar pathways from the six tested mechanisms. One different mechanism was the requirement of L-type calcium channels for memantine’s neuroprotection, but not for 4R’s. When we compare the doses at which they both induce neuroprotection, we observe that 4R has a broad range of neuroprotection (2 μM–40 μM), but memantine has a shorter range (1 μM–3 μM). Checked mark = required in the process, X = not required.

A large-scale patient study on the efficacy and safety of memantine showed AD clinical heterogeneity as a challenge that cannot be treated with a single drug and recommended a multidrug treatment approach for this devastating disease (McShane et al., 2019). The results in the present study, summarized in Fig. 10, expand on memantine’s nicotinic mechanisms of neuroprotection. These are comparable to other neuroprotective drugs with nicotinic mechanisms such as 4R. In conclusion, this report provides new evidence regarding the nicotinic neuroprotective mechanism of memantine and introduces the 4R cembranoid as an alternative to treat Alzheimer’s. 4R has a similar neuroprotective nicotinic mechanism of action as memantine. However, memantine is neurotoxic at higher concentrations (see Fig. 1), most probably because it blocks the subsynaptic NMDA receptors. Under the same conditions, the neuroprotection by 4R, which is entirely dependent on the nicotinic receptors, extends from 2 to 40 μM.

## 5. Future directions

Although the results presented do not directly relate to the clinical use of memantine, they suggest that 4R could be a better choice to prevent neurodegeneration caused by AD since they share similar mechanisms and, unlike memantine, higher concentrations of 4R do not inhibit neuroprotection. Future experiments of neuroprotection involving 4R and animal models of Alzheimer’s should answer whether 4R could decrease neurodegeneration involving tau and Aβ.

## Supplementary Material

Supplementar Figure 1
