# Supplementary figures and images for: Memantine has a nicotinic neuroprotective pathway in acute hippocampal slices after an NMDA insult
Source: Toxicol In Vitro. Author manuscript; Available in PMC 2023 Mar 20. (PMC10026604; doi:10.1016/j.tiv.2022.105453)

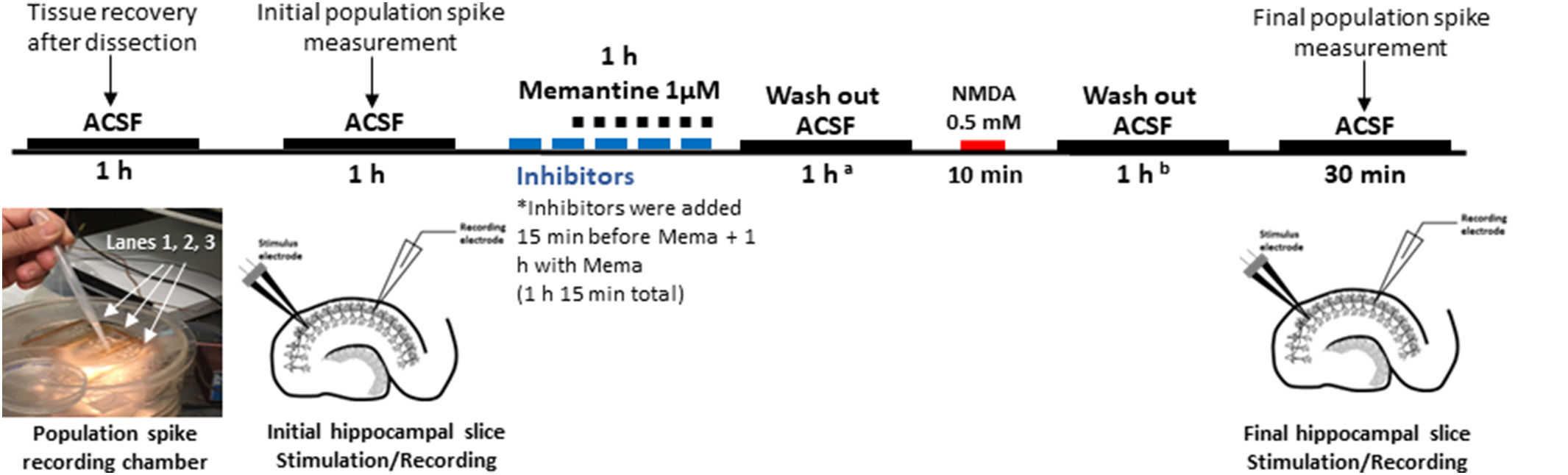

Supplement: Supplementar Figure 1 [file NIHMS1873316-supplement-Supplementar_Figure_1.jpg]
